# Supplementary material for: Boreal conifers maintain carbon uptake with warming despite failure to track optimal temperatures
Source: Nat Commun. 2023 Aug 3;14:4667. doi: 10.1038/s41467-023-40248-3 (PMC10400668; doi:10.1038/s41467-023-40248-3)
Supplement: Supplementary file 1 — Supplementary Information [file 41467_2023_40248_MOESM1_ESM.pdf]

SUPPLEMENTARY INFORMATION

Boreal conifers maintain carbon uptake with warming despite failure to track optimal temperatures

Mirindi Eric Dusenge [1,2,3,*], Jeffrey M. Warren [4], Peter B. Reich[5, 6, 7], Eric J. Ward [8], Bridget K. Murphy [3, 9, 10], Artur Stefanski [5], Raimundo Bermudez [5], Marisol Cruz [11], David A. McLennan [4], Anthony W. King [4], Rebecca A. Montgomery [5], Paul J. Hanson [4], Danielle A. Way [3, 12, 13, 14, *]

[1] Department of Biology, Mount Allison University, Sackville, New Brunswick, E4L 1E4, Canada
[2] Western Centre for Climate Change, Sustainable Livelihoods and Health, Department of Geography and Environment, The University of Western Ontario, London, Ontario, N6G 2V4, Canada
[3] Department of Biology, The University of Western Ontario, London, Ontario, N6A 3K7, Canada
[4] Climate Change Science Institute and Environmental Sciences Division, Oak Ridge National Laboratory, Oak Ridge, Tennessee 37830, USA
[5] Department of Forest Resources, University of Minnesota, Saint Paul, Minnesota 55108, USA
[6] Hawkesbury Institute for the Environment, University of Western Sydney, Penrith, New South Wales 2753, Australia
[7] Institute for Global Change Biology, and School for the Environment and Sustainability, University of Michigan, Ann Arbor, Michigan 48109, USA
[8] US Geological Survey, Wetland and Aquatic Research Center, Lafayette, Louisiana, USA
[9] Department of Biology, University of Toronto Mississauga, Mississauga, Ontario, L5L 1C6, Canada
[10] Graduate Program in Cell and Systems Biology, University of Toronto, Toronto, Ontario, M5S 3B2, Canada
[11] Departamento de Ciencias Biologicas, Universidad de Los Andes, Bogota, Colombia
[12] Division of Plant Sciences, Research School of Biology, The Australian National University, Canberra, ACT 2601, Australia
[13] Nicholas School of the Environment, Duke University, Durham, North Carolina 27708, USA
[14] Environmental and Climate Sciences Department, Brookhaven National Laboratory, Upton, New York 11973, USA

*Contact authors: mdusenge@uwo.ca; danielle.way@anu.edu.au

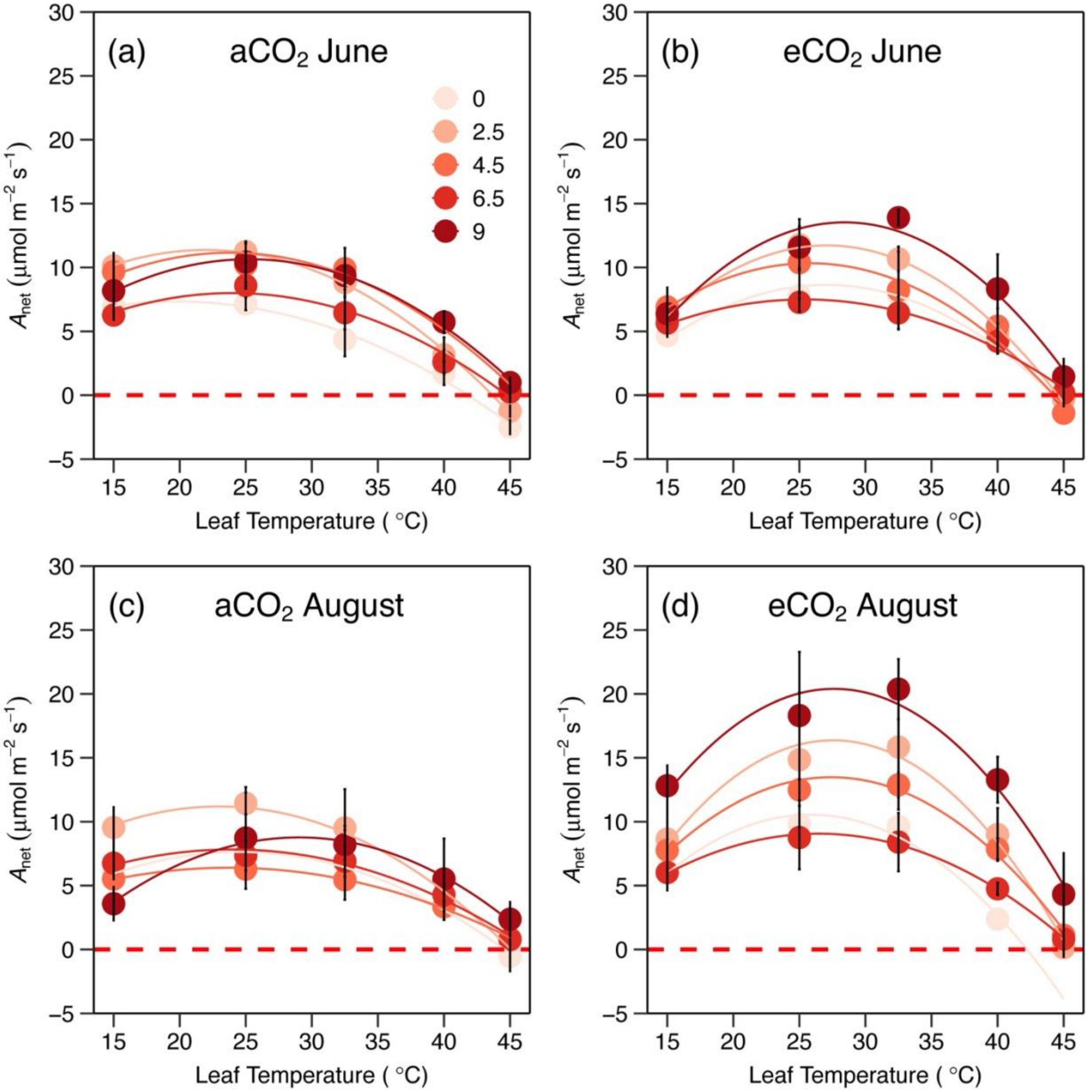

Supplementary Figure 1 | Temperature response curve of net photosynthesis for tamarack. (a) and (c) panels are responses for ambient CO_2 (aCO_2) for June and August, respectively, while (b) and (d) are panels for elevated CO_2 (eCO_2) for June and August, respectively. The different red color gradients represent different warming treatments (0, +2.25, +4.5, +6.75, and +9 °C). The long-dashed, horizontal line indicates the x-axis at the origin. Each data point represents the mean value of biologically independent trees measured in each plot: (a) aCO_2 June: n = 3, 3, 3, 3, and 3 biologically independent trees for +0, +2.25, +4.5, +6.75, and +9 °C, respectively; (b) eCO_2 June: n = 1, 3, 2, 4, and 3 biologically independent trees for +0, +2.25, +4.5, +6.75, and +9 °C, respectively; (c) aCO_2 August: n = 2, 2, 1, 2, and 2 biologically independent trees for +0, +2.25, +4.5, +6.75, and +9 °C, respectively; (d) eCO_2 August: n = 1, 2, 2, 2, and 2 biologically independent trees for +0, +2.25, +4.5, +6.75, and +9 °C, respectively. Mean ± SE.

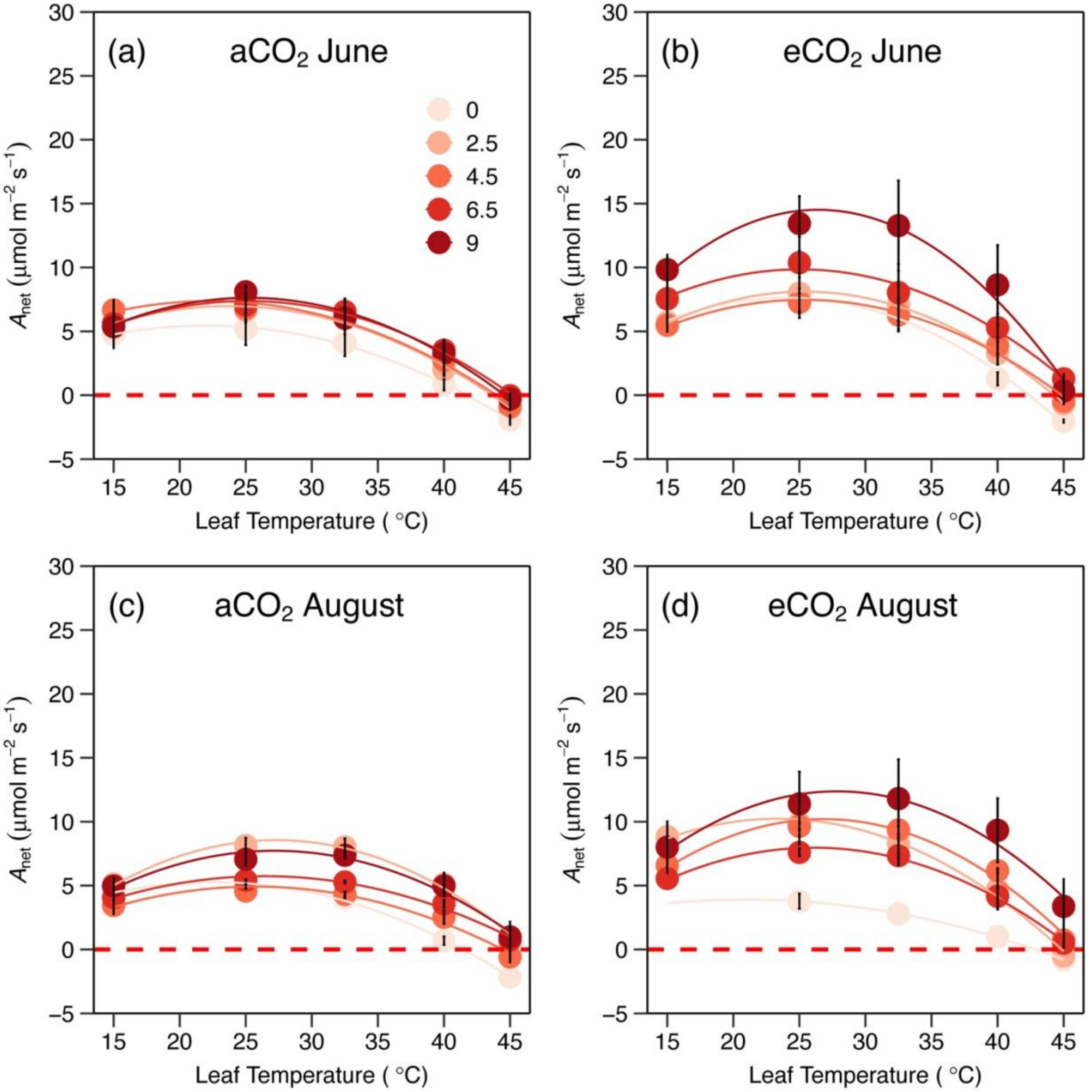

Supplementary Figure 2 | Temperature response curve of net photosynthesis for black spruce. (a) and (c) panels are responses for ambient CO_2 (aCO_2) for June and August, respectively, while (b) and (d) are panels for elevated CO_2 (eCO_2) for June and August, respectively. The different red color gradients represent different warming treatments (0, +2.25, +4.5, +6.75, and +9 °C). The long-dashed, horizontal line indicates the x-axis at the origin. Each data point represents the mean value of biologically independent trees measured in each plot: (a) aCO_2 June: n = 3, 3, 3, 3, and 4 biologically independent trees for +0, +2.25, +4.5, +6.75, and +9 °C, respectively; (b) eCO_2 June: n = 3, 3, 4, 3, and 3 biologically independent trees for +0, +2.25, +4.5, +6.75, and +9 °C, respectively; (c) aCO_2 August: n = 2, 2, 2, 2, and 2 biologically independent trees for +0, +2.25, +4.5, +6.75, and +9 °C, respectively; (d) eCO_2 August: n = 2, 2, 2, 2, and 2 biologically independent trees for +0, +2.25, +4.5, +6.75, and +9 °C, respectively. Mean ±SE.

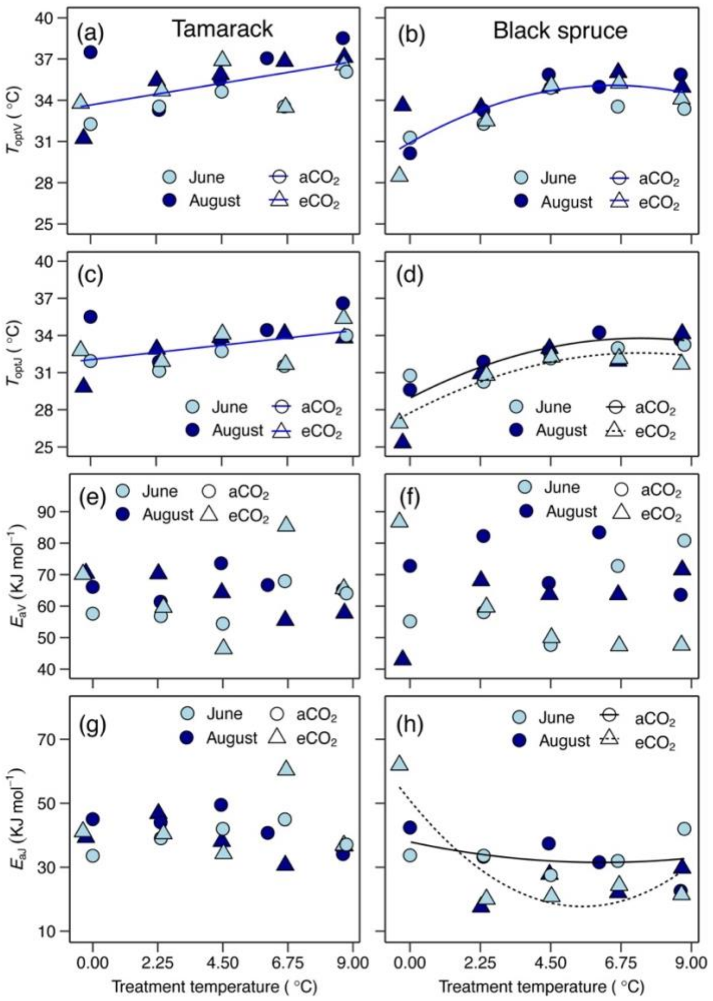

Supplementary Figure 3 | Impact of temperature and CO_2 treatments on the thermal sensitivity of the photosynthetic biochemical processes in tamarack (a) and black spruce (b). Thermal optima of a, b) the maximum Rubisco carboxylation rate – V_{cmax} (T_{optV}, ˚C) and c, d) the maximum rate of electron transport – J_{max} (T_{optJ}, ˚C); and activation energies of e,f) V_{cmax} (E_{aV}) and g, h) J_{max} (E_{aJ}). Symbol colours represent the month in which measurements were taken (June = light blue; August = dark blue). Symbol shapes represent CO_2 treatments (circle = ambient CO_2 – aCO_2; triangles = elevated CO_2 – eCO_2). A mixed-effects regression model was used to analyze the data where warming and elevated CO_2 treatment were the fixed effects, and the month in which the campaign was done was the random effect. The statistical test was one-sided since it was done to evaluate whether warming and elevated CO_2 increase photosynthetic thermal sensitivity parameters. Blue lines (a,b,c) (a: y = 0.35 x + 33.7; p = 0.010; b: y = 1.3 x^2 -0.1 x + 31; p = 0.0004, and c: y = 0.26 x + 32; p = 0.029) represent the overall regression line when there is no effect of CO_2 on the slope and intercept, while in (d and h) the solid (d: y = -0.09 x^2 + 1.31 x + 28.9; p = 0.0065; h: y = 0.17 x^2 -2.1 x+ 37.9; p = 0.56) and short-dashed (d: y = -0.09 x^2 + 1.31 x + 27.7; p = 0.0065; h: y = 1 x^2 -11.9 x+ 51; p = 0.045) lines represent ambient and elevated CO_2 treatment, respectively. In (e: y = 0.003x + 63.4, p = 0.99 and y = 0.003x + 64.4, p = 0.99, for ambient and elevated CO_2 treatments, respectively), (f: y = 0.045x + 68.2, p = 0.96, and y = 0.045x + 59.9, p = 0.96, for ambient and elevated CO_2 treatments, respectively), (g: y = -0.24x + 42, p = 0.65 and -0.24x + 42, p = 0.65, for ambient and elevated CO_2 treatments, respectively), E_{av} and E_{aJ} did not significantly change with treatments. Each data point represents the mean value of trees measured in each plot (n = 1-4 trees/plot). Significance threshold: $p < 0.05$. Further details on statistical analyses for this figure can be found in Supplementary Table 1.

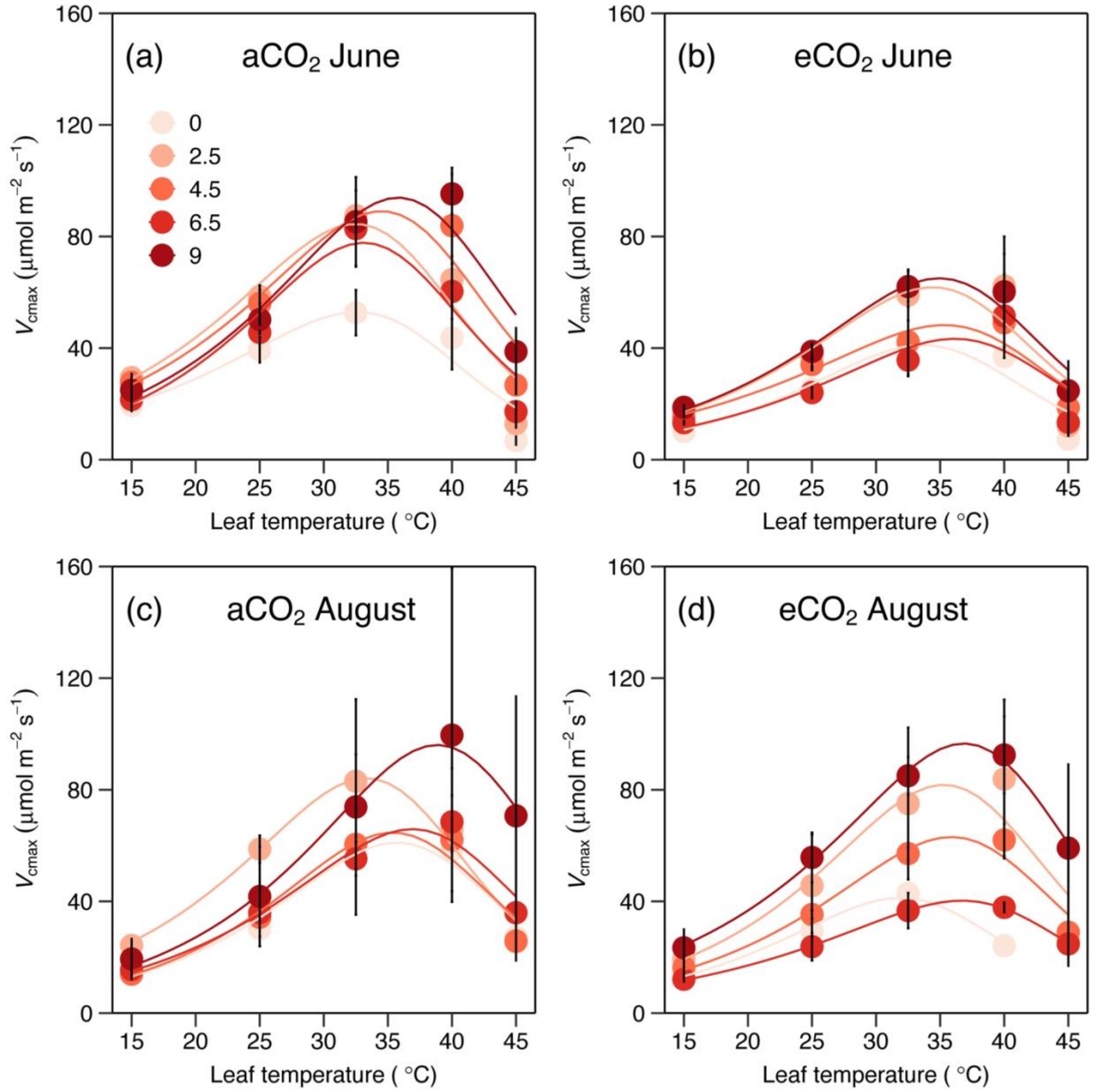

Supplementary Figure 4 | Temperature response curve of the maximum Rubisco carboxylation (V_{cmax}) rate for tamarack. (a) and (c) panels are responses for ambient CO_2 (aCO_2) for June and August, respectively, while (b) and (d) are panels for elevated CO_2 (eCO_2) for June and August, respectively. The different red color gradients represent different warming treatments (0, +2.25, +4.5, +6.75, and +9 °C). Each data point represents the mean value of biologically independent trees measured in each plot: (a) aCO_2 June: n = 3, 3, 3, 3, and 3 biologically independent trees for +0, +2.25, +4.5, +6.75, and +9 °C, respectively; (b) eCO_2 June: n = 1, 3, 2, 4, and 3 biologically independent trees for +0, +2.25, +4.5, +6.75, and +9 °C, respectively; (c) aCO_2 August: n = 2, 2, 1, 2, and 2 biologically independent trees for +0, +2.25, +4.5, +6.75, and +9 °C, respectively; (d) eCO_2 August: n = 1, 2, 2, 2, and 2 biologically independent trees for +0, +2.25, +4.5, +6.75, and +9 °C, respectively. Mean ±SE.

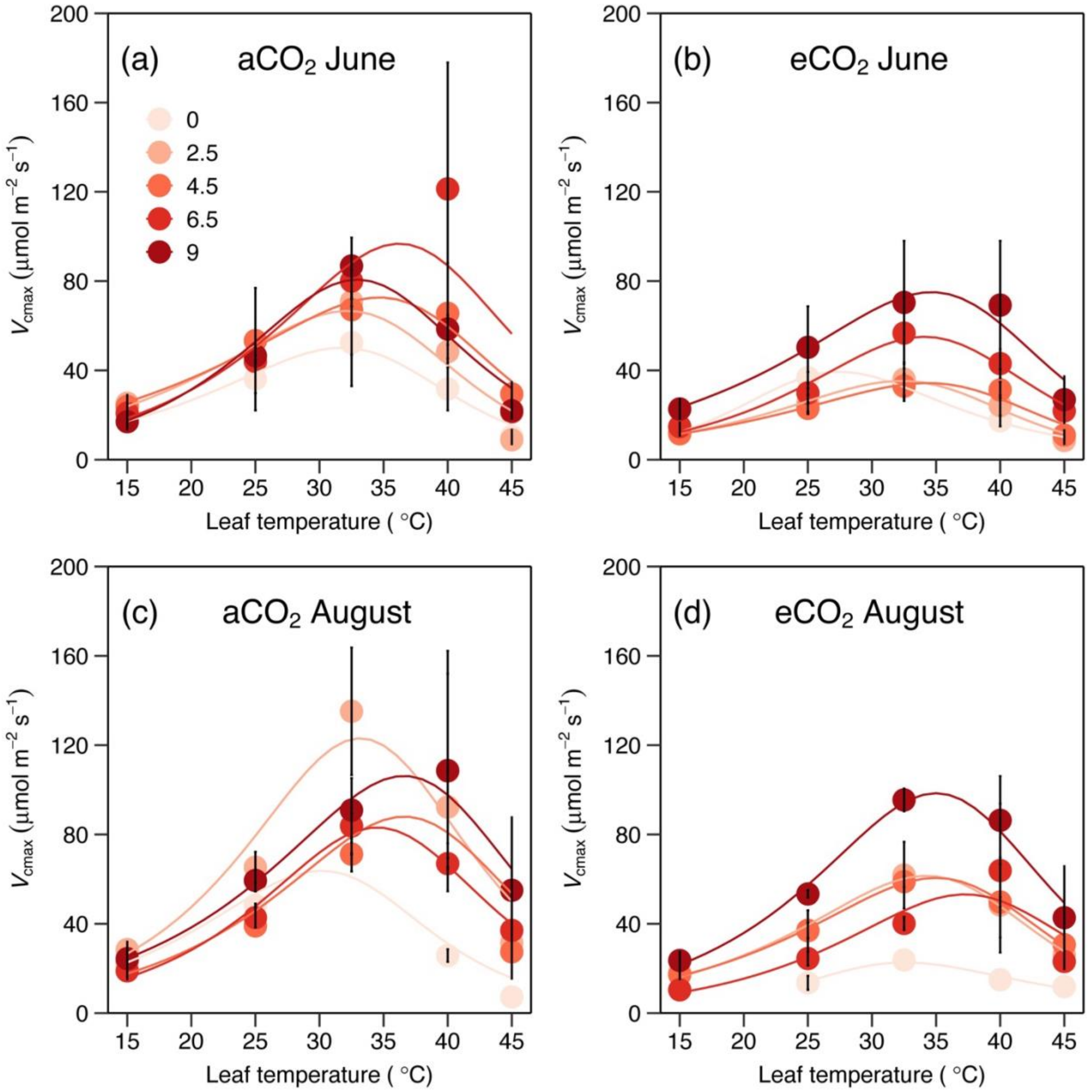

Supplementary Figure 5 | Temperature response curve of the maximum Rubisco carboxylation rate (V_{cmax}) for black spruce. (a) and (c) panels are responses for ambient CO_2 (aCO_2) for June and August, respectively, while (b) and (d) are panels for elevated CO_2 (eCO_2) for June and August, respectively. The different red color gradients represent different warming treatments (0, +2.25, +4.5, +6.75, and +9 °C). Each data point represents the mean value of biologically independent trees measured in each plot: (a) aCO_2 June: n = 3, 3, 3, 3, and 4 biologically independent trees for +0, +2.25, +4.5, +6.75, and +9 °C, respectively; (b) eCO_2 June: n = 3, 3, 4, 3, and 3 biologically independent trees for +0, +2.25, +4.5, +6.75, and +9 °C, respectively; (c) aCO_2 August: n = 2, 2, 2, 2, and 2 biologically independent trees for +0, +2.25, +4.5, +6.75, and +9 °C, respectively; (d) eCO_2 August: n = 2, 2, 2, 2, and 2 biologically independent trees for +0, +2.25, +4.5, +6.75, and +9 °C, respectively. Mean ±SE.

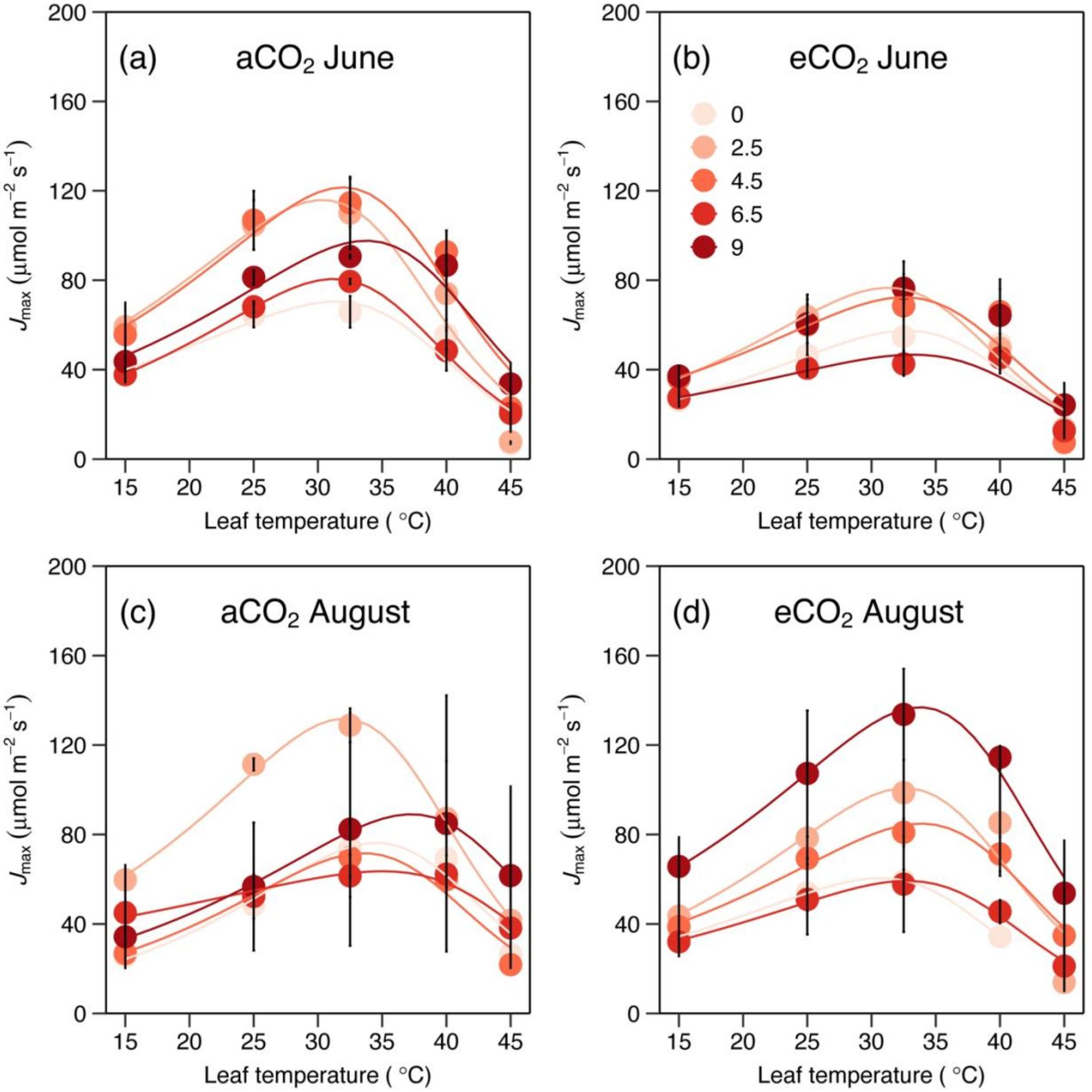

Supplementary Figure 6 | Temperature response curve of the maximum electron transport rate (J_{max}) for tamarack. (a) and (c) panels are responses for ambient CO_2 (aCO_2) for June and August, respectively, while (b) and (d) are panels for elevated CO_2 (eCO_2) for June and August, respectively. The different red color gradients represent different warming treatments (0, +2.25, +4.5, +6.75, and +9 °C). Each data point represents the mean value of biologically independent trees measured in each plot: (a) aCO_2 June: n = 3, 3, 3, 3, and 3 biologically independent trees for +0, +2.25, +4.5, +6.75, and +9 °C, respectively; (b) eCO_2 June: n = 1, 3, 2, 4, and 3 biologically independent trees for +0, +2.25, +4.5, +6.75, and +9 °C, respectively; (c) aCO_2 August: n = 2, 2, 1, 2, and 2 biologically independent trees for +0, +2.25, +4.5, +6.75, and +9 °C, respectively; (d) eCO_2 August: n = 1, 2, 2, 2, and 2 biologically independent trees for +0, +2.25, +4.5, +6.75, and +9 °C, respectively. Mean ±SE.

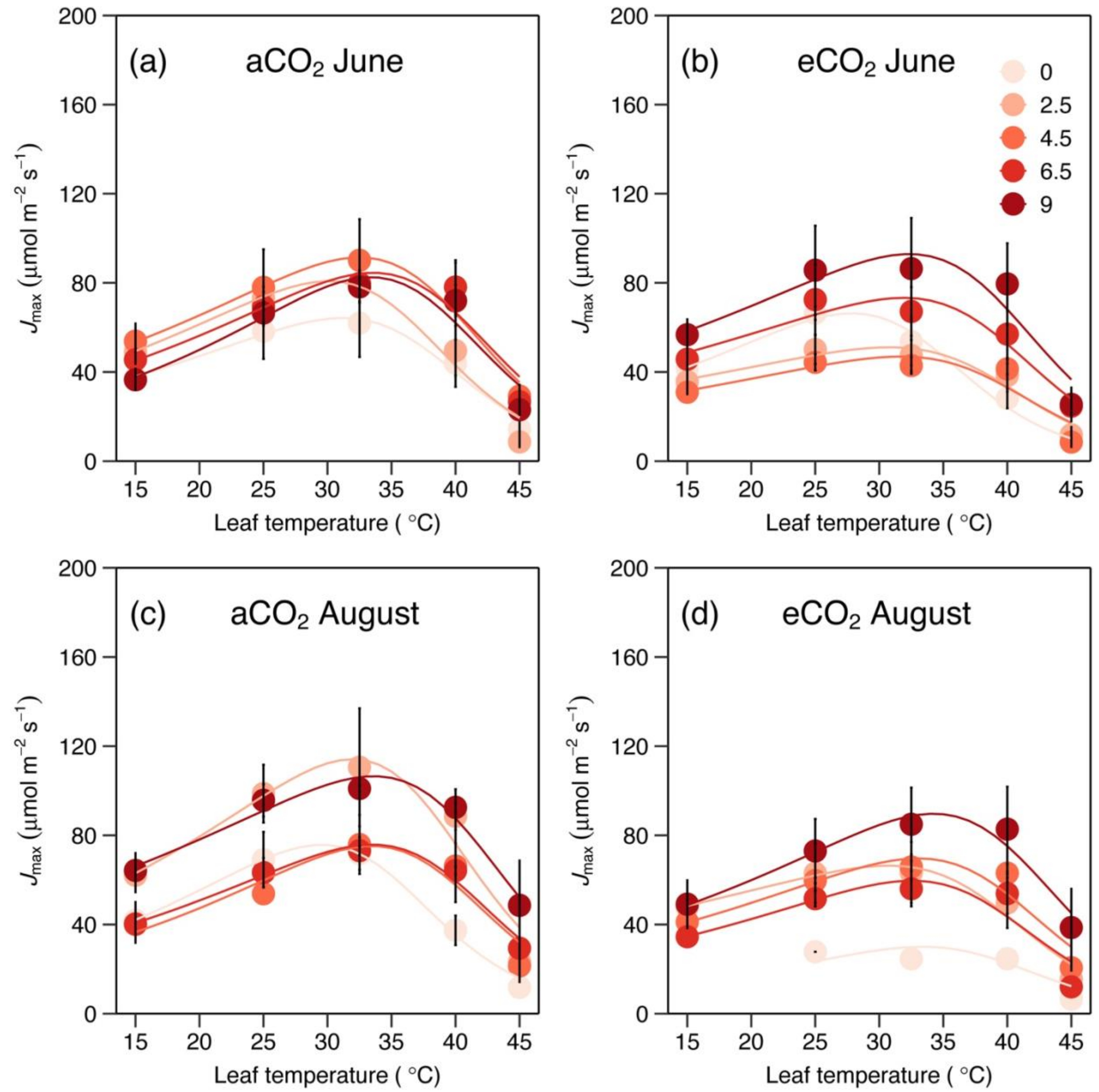

Supplementary Figure 7 | Temperature response curve of the maximum electron transport rate (J_{max}) for black spruce. (a) and (c) panels are responses for ambient CO_2 (aCO_2) for June and August, respectively, while (b) and (d) are panels for elevated CO_2 (eCO_2) for June and August, respectively. The different red color gradients represent different warming treatments (0, +2.25, +4.5, +6.75, and +9 °C). Each data point represents the mean value of biologically independent trees measured in each plot: (a) aCO_2 June: n = 3, 3, 3, 3, and 4 biologically independent trees for +0, +2.25, +4.5, +6.75, and +9 °C, respectively; (b) eCO_2 June: n = 3, 3, 4, 3, and 3 biologically independent trees for +0, +2.25, +4.5, +6.75, and +9 °C, respectively; (c) aCO_2 August: n = 2, 2, 2, 2, and 2 biologically independent trees for +0, +2.25, +4.5, +6.75, and +9 °C, respectively; (d) eCO_2 August: n = 2, 2, 2, 2, and 2 biologically independent trees for +0, +2.25, +4.5, +6.75, and +9 °C, respectively. Mean ± SE.

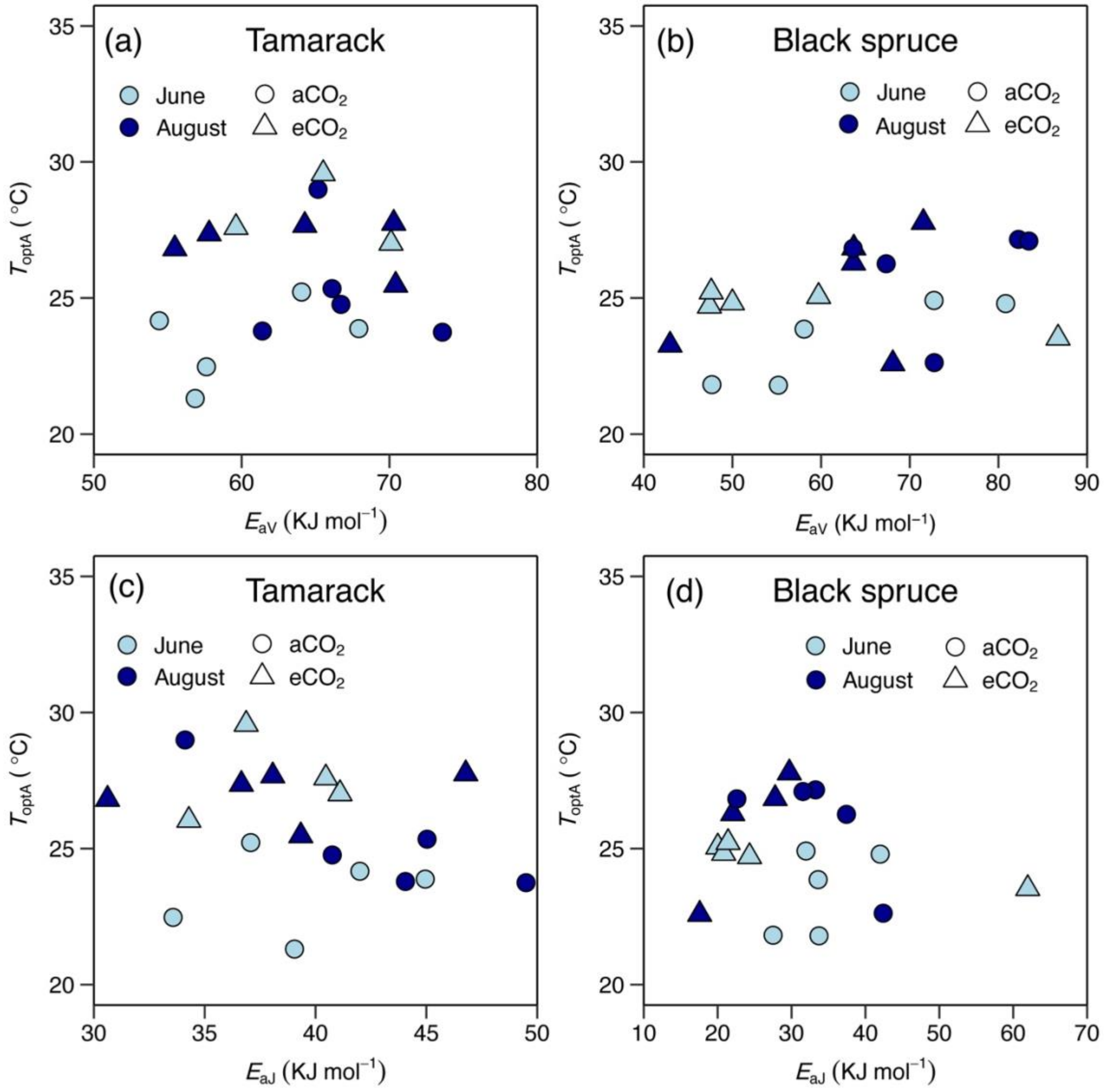

Supplementary Figure 8 | The temperature optimum of net photosynthesis measured at growth CO_2 (T_{optA}, °C) as a function of the activation energy of a, b) the maximum Rubisco carboxylation rate (E_{aV}, kJ mol^{-1}); c, d) the maximum electron transport rate (E_{aJ}, kJ mol^{-1}) in tamarack (a, c) and black spruce (b, d). Symbol shapes represent CO_2 treatments (ambient CO_2 – aCO_2 = circle; elevated CO_2 – eCO_2 = triangle). Symbol colours represent the month in which measurements were taken (June = light blue; August = dark blue). A mixed-effects regression model was used to analyze the data where warming and elevated CO_2 treatment were the fixed effects, and the month in which the campaign was done was the random effect. The statistical test was one-sided since it was done to evaluate whether there is a positive relationship among the thermal optimum of net photosynthesis and activation energies of the underlying biochemical processes. The T_{optA} was not significantly related to either E_{aV} (a: aCO_2 - y = 0.036x + 22.1, $p = 0.44$; eCO_2 - y = 0.036x + 24.9, $p = 0.44$; b: aCO_2 - y = 0.06x + 20.7, $p = 0.091$; eCO_2 - y = 0.06x + 21.4, $p = 0.091$) or E_{aJ} (c: aCO_2 - y = -0.025x + 25.4, $p = 0.67$; eCO_2 - y = -0.025x + 28.1, $p = 0.67$, d: aCO_2 - y = -0.03x + 25.7, $p = 0.55$; eCO_2 - y = -0.03x + 26, $p = 0.55$) in either species. Each data point represents the mean value of trees measured in each plot (n = 1-4 trees/plot). Significance threshold: $p < 0.05$. Further details on statistical analyses for this figure can be found in Supplementary Table 2.

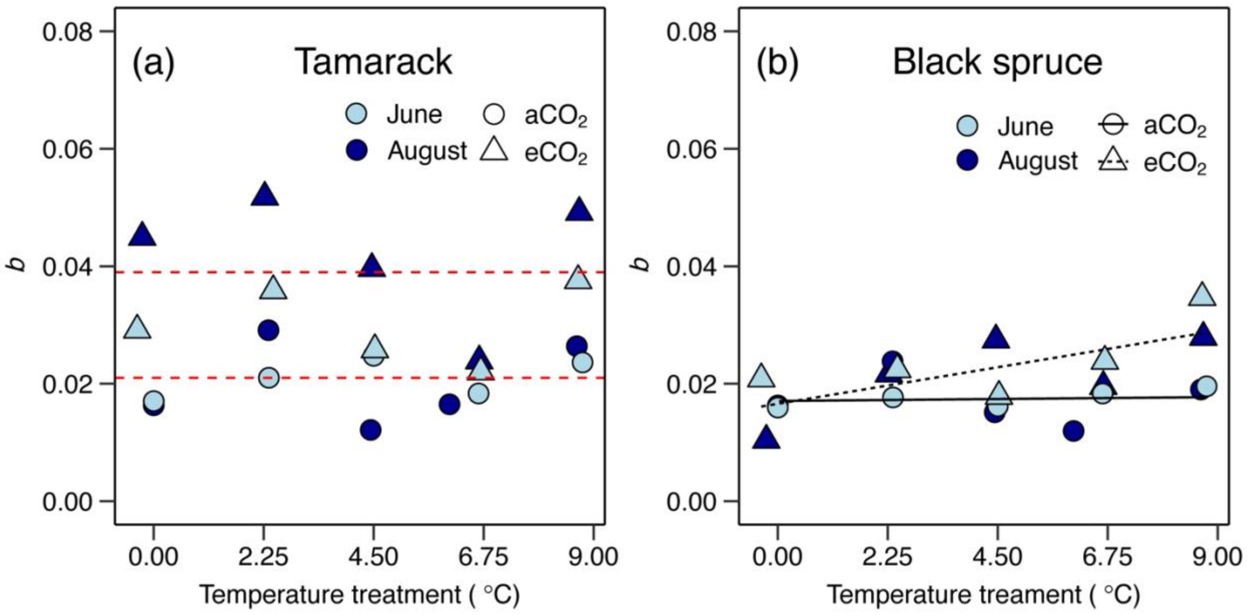

Supplementary Figure 9 | Impact of temperature and CO_2 treatments on the *b* parameter representing the breadth of the temperature response curve of net photosynthesis in tamarack (a) and black spruce (b). The *b* parameter was estimated from the temperature response curve of net photosynthesis measured at growth CO_2 using Equation 2 (see Methods). Symbol colours represent the month in which measurements were taken (June = light blue; August = dark blue). Symbol shapes represent CO_2 treatments (circle = ambient CO_2 – aCO_2; triangles = elevated CO_2 – eCO_2). A mixed-effects regression model was used to analyze the data where warming and elevated CO_2 treatment were the fixed effects, and the month in which the campaign was done was the random effect. The statistical test was two-sided. Regression lines in (b): the solid (y = 7.406e-05 x + 0.017, $p = 0.86$) and short-dashed (y = 0.0013x + 0.016, $p = 0.046$) lines represent ambient and elevated CO_2 treatments, respectively; In (a), red long-dashed (ambient CO_2 – y = 1.62e-05x + 0.02, $p = 98$; elevated CO_2 – y = 1.62e-05x + 0.039, $p = 98$) lines represent the overall mean value across all treatment temperatures. Each data point represents the mean value of trees measured in each plot (n = 1-4 trees/plot). Significance threshold: $p < 0.05$. Further details on statistical analyses for this figure can be found in Supplementary Table 1.

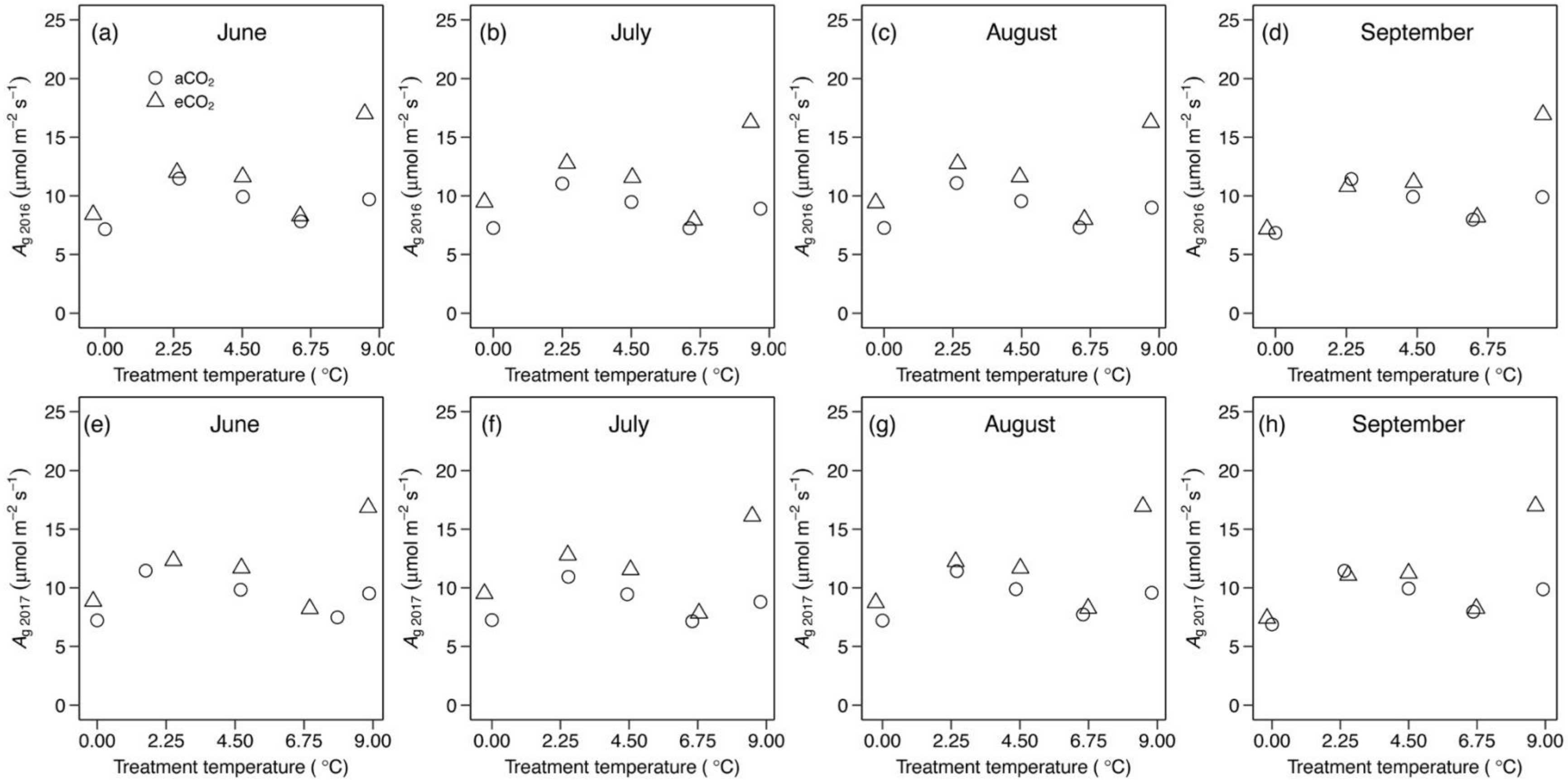

Supplementary Figure 10 | Impact of temperature and CO_2 treatments on net photosynthesis rate estimated at monthly mean growth temperature (9 am – 3 pm) throughout the 2016 and 2017 growth season for tamarack. The estimates of net photosynthesis rates were obtained by replacing in Equation 2 the average plot values of b, T_{optA} and A_{opt} for each species. Symbol shapes represent CO_2 treatments (circle = ambient CO_2 – aCO_2; triangles = elevated CO_2 – eCO_2). Each data point represents a plot-level value. An Analysis of Covariance (ANCOVA) test was used to analyze the data, where temperature was a covariate and CO_2 treatment as a fixed factor. The statistical test was one-sided since it was done to evaluate whether warming and elevated CO_2 stimulate A_g in 2016 and 2017. Details on statistical analyses for each figure panel are provided in the Supplementary Table 5.

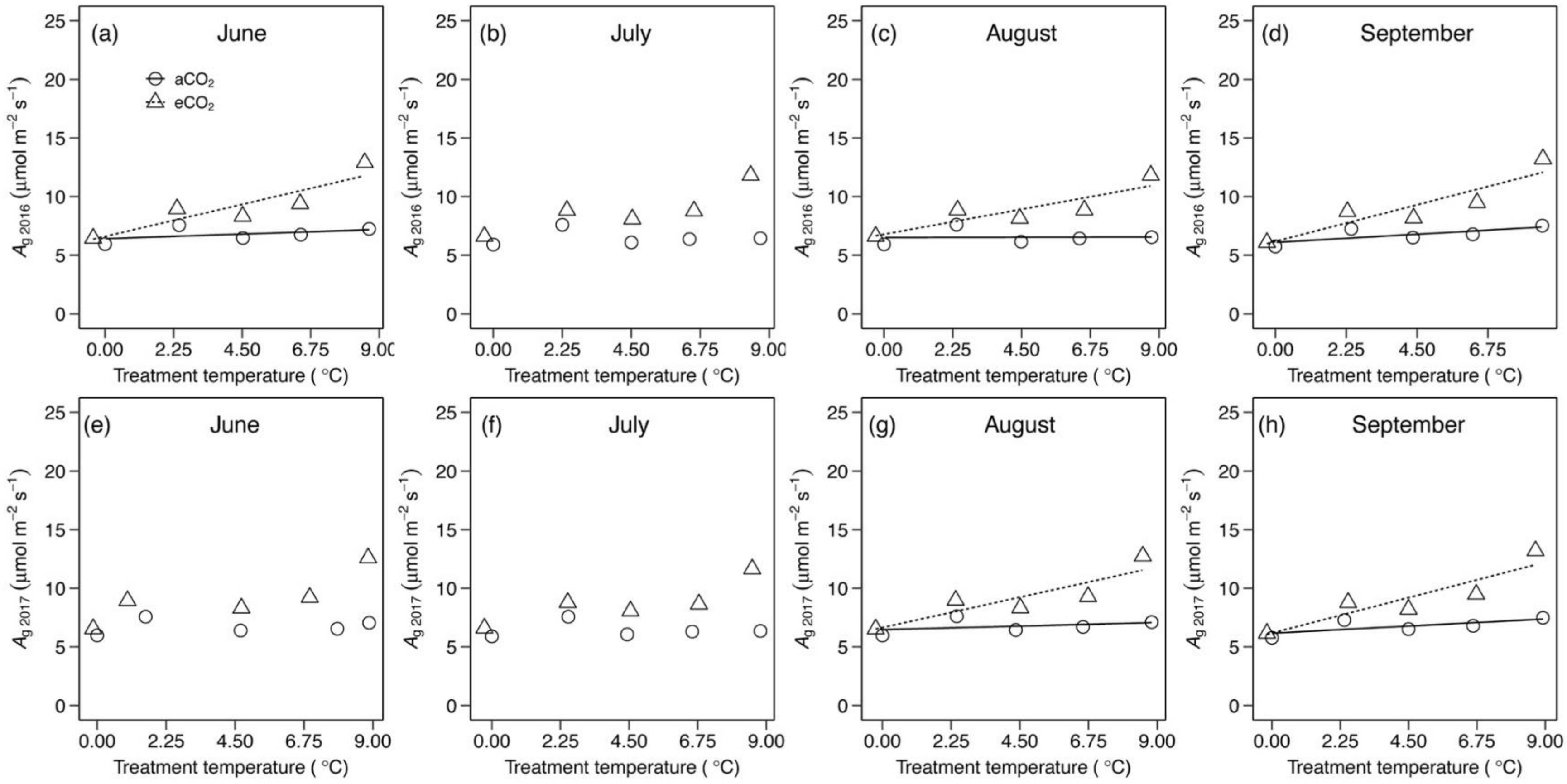

Supplementary Figure 11 | Impact of temperature and CO_2 treatments on net photosynthesis rate modeled at monthly mean growth temperature (9 am – 3 pm) throughout the 2016 and 2017 growth season for black spruce. The modeled net photosynthesis rates were obtained by replacing in Equation 2 the average plot values of b, T_{optA} and A_{opt} for each species. Symbol shapes represent CO_2 treatments (circle = ambient CO_2 – aCO_2; triangles = elevated CO_2 – eCO_2). Lines represent regression lines: the solid and short-dashed lines are for ambient and elevated CO_2 treatments, respectively. Each data point represents a plot -level value. An analysis of covariance (ANCOVA) test was used to analyze the data where temperature was a covariate and CO_2 treatment as a fixed factor. The statistical test was one-sided since it was done to evaluate whether warming and elevated CO_2 stimulate A_g. Details on statistical analyses for each figure panel are provided in the Supplementary Table 5.

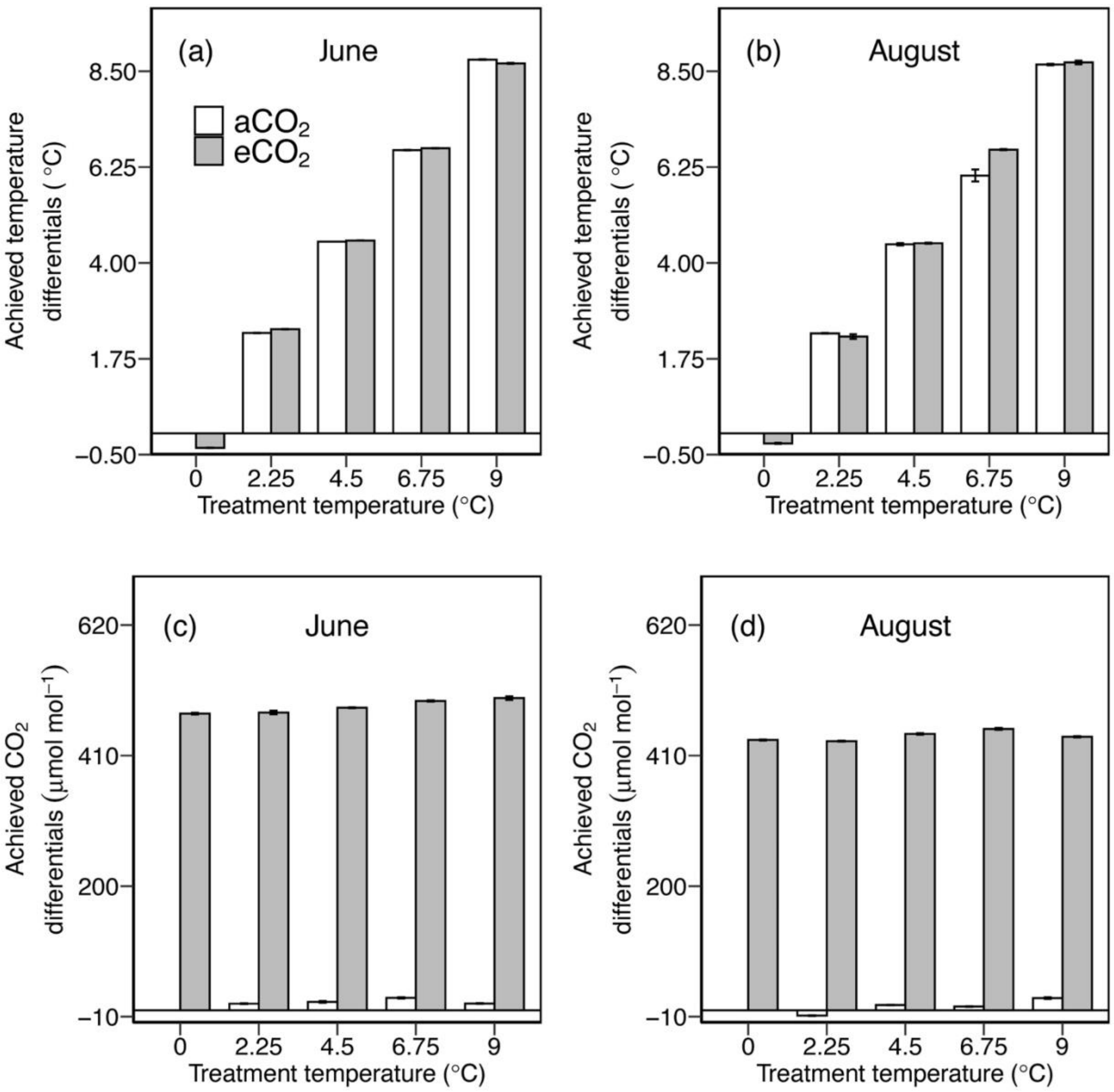

Supplementary Figure 12 | Environmental data for the ten enclosures. (a, b) Achieved temperature and (c, d) CO_2 differentials for June and August. The temperature and atmospheric CO_2 differentials were calculated as average of ten days preceding each measurement day, and these ten-days averaged differentials for each enclosure were further averaged across all measurement days in each month which resulted in n = 11 and 13 measurement days in June and August per each enclosure, respectively. Means ± SE. Colors represent CO_2 treatments (ambient CO_2 – aCO_2= white; Elevated CO_2 – eCO_2= gray).

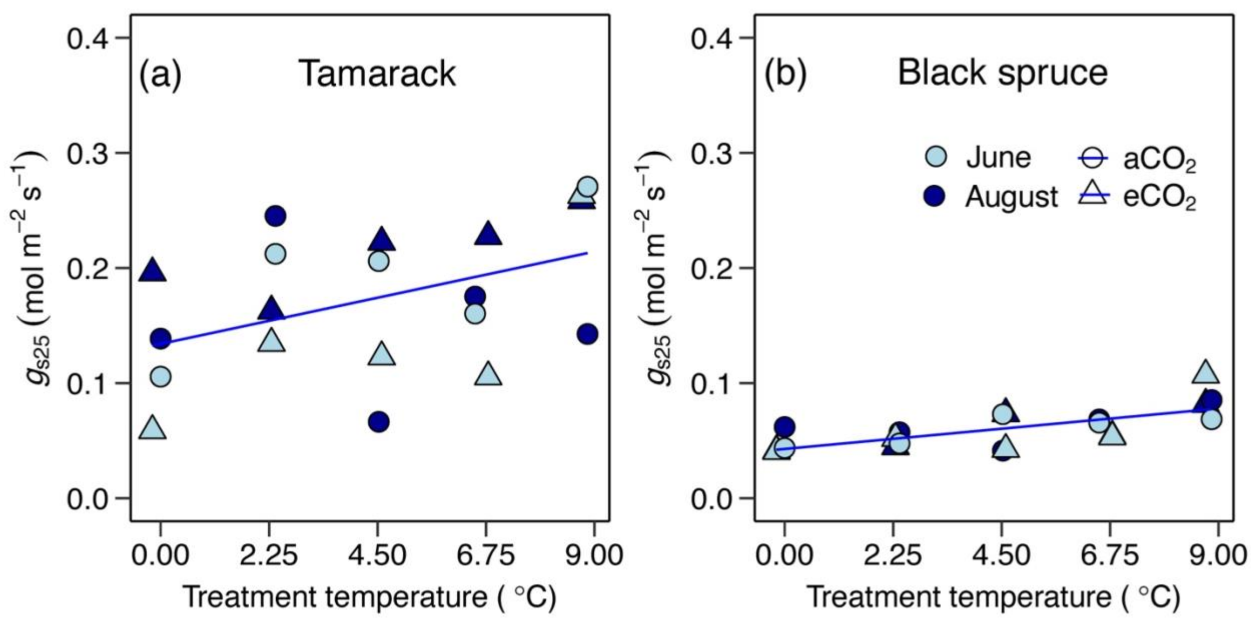

Supplementary Figure 13 | Impact of temperature and CO_2 treatments on stomatal conductance at a leaf temperature of 25 °C (g_{s25}; mol m^{-2} s^{-1}) in tamarack (a) and black spruce (b). Symbol colours represent the month in which measurements were taken (June = light blue; August = dark blue). Symbol shapes represent CO_2 treatments (circle = ambient CO_2 – aCO_2; triangles = elevated CO_2 – eCO_2). A mixed-effects regression model was used to analyze the data where warming and elevated CO_2 treatment were the fixed effects, and the month in which the campaign was done was the random effect. The statistical test was two-sided. The blue line (a: y = 0.01x + 0.13, p = 0.056; b: y = 0.003x + 0.049, p = 0.00076) represents the overall regression line when there is no effect of CO_2 on the slope and intercept. Each data point represents the mean value of trees measured in each plot (n = 1-4 trees/plot). Significance threshold: $p < 0.05$. Further details on statistical analyses for this figure can be found in Supplementary Table 1.

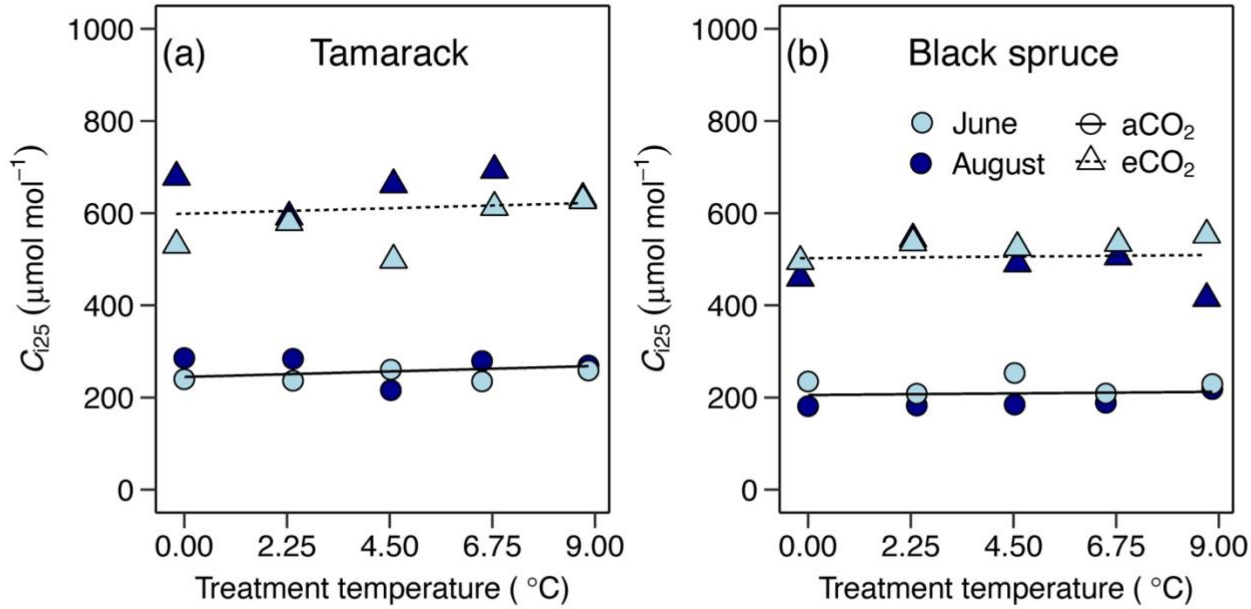

Supplementary Figure 14 | Impact of temperature and CO_2 treatments on intercellular CO_2 concentration at a leaf temperature of 25 °C (C_i; µmol m^{-2} s^{-1}) in tamarack (a) and black spruce (b). Symbol colours represent the month in which measurements were taken (June = light blue; August = dark blue). Symbol shapes represent CO_2 treatments (circle = ambient CO_2 – aCO_2; triangles = elevated CO_2 – eCO_2). A mixed-effects regression model was used to analyze the data where warming and elevated CO_2 treatment were the fixed effects, and the month in which the campaign was done was the random effect. The statistical test was two-sided. Regression lines: the solid (a: y = 2.6x + 244.5, p = 0.45; b: y = 0.77x + 205.3, p = 0.76) and short-dashed (a: y = 2.6x + 599, p = 0.45; b: y = 0.77x + 502, p = 0.76) lines represent ambient and elevated CO_2 treatments, respectively. Each data point represents the mean value of trees measured in each plot (n = 1-4 trees/plot). Significance threshold: $p < 0.05$. Further details on statistical analyses for this figure can be found in Supplementary Table 1.

Supplementary Table 1 | Parameter estimates for the adequate models explaining the responses of temperature sensitivity parameters of photosynthesis to temperature and CO_2 treatments in tamarack and black spruce.

Tamarack									
Trait	Slope (Temperature and aCO_2)	Intercept (eCO_2)	CO_2 treatment	Temperature* CO_2	Month intercept		Fixed effect (marginal R^2)	Random effect R^2	Total (conditional) R^2
					Variance for random intercept (Month)	Residual variance			
T_{optV}	$p = 0.010$		$p = 0.99$	$p = 0.68$	$p = 0.31$		0.33	-	0.33
	0.35±0.12	33.7±0.7	33.7±0.7						
T_{optJ}	$p = 0.029$		$p = 0.66$	$p = 0.61$	$p = 0.59$		0.26	-	0.26
	0.26±0.1	32.2±0.6	31.9±0.6						
E_{aV}	$p = 0.99$		$p = 0.77$	$p = 0.37$	$p = 0.99$		0.005	-	0.005
	0.003±0.6	63.4±3.9	64.4±3.9						
E_{aJ}	$p = 0.65$		$p = 0.86$	$p = 0.95$	$p = 0.99$		0.015	-	0.015
	-0.24±0.5	42±3.1	41.5±3.1						
T_{optA}	$p = 0.021$		$p = 0.00036$	$p = 0.32$	$p = 0.74$		0.61	-	0.61
	0.26±0.1	23.2±0.64	26.0±0.6						
b	$p = 0.98$		$p = 0.00092$	$p = 0.51$	$p = 0.57$		0.48	-	0.48
	1.62e-05±6.2e-04	0.02±0.004	0.039±0.004						
A_{opt}	$p = 0.27$		$p = 0.045$	$p = 0.46$	$p = 0.99$		0.26	-	0.26
	0.26±0.22	7.9±1.4	10.89±1.4						
A_g	$p = 0.35$		$p = 0.04$	$p = 0.42$	$p = 0.99$		0.26	-	0.26
	0.2±0.2	7.9±1.3	10.79±1.3						
g_{s25}	$p = 0.056$		$p = 0.91$	$p = 0.41$	$p = 0.41$		0.2	-	0.2
	0.01±0.004	0.13±0.02	0.13±0.02						
C_{i25}	$p = 0.45$		$p < 0.0001$	$p = 0.46$	$p = 0.052$		0.94	-	0.94
	2.6±3.4	244.5±21.5	598.8±21.37						

Black spruce										
Trait	Slope (Temperature and aCO_2)	Slope (Temperature^2 and aCO_2)	Intercept (AC)	CO_2 treatment	Temperature* CO_2	Month intercept		Fixed effect (marginal R^2)	Random effect R^2	Total (Conditional) R^2
						Variance for random intercept (Month)	Residual variance			
T_{optV}	$p = 0.0004$	$p = 0.007$		$p = 0.52$	$p = 0.89$	$p = 0.24$		0.68	-	0.68
	1.3±0.29	-0.1±0.03	30.7±0.6	31±0.5						
T_{optJ}	$p < 0.0001$	$p = 0.0065$		$p = 0.024$	$p = 0.074$	$p = 0.99$		0.81	-	0.81
	1.31±0.3	-0.09±0.03	28.9±0.5	27.7±0.5						
E_{aV}	$p = 0.96$			$p = 0.18$	$p = 0.32$	$p = 0.72$		0.1	-	0.1
	0.045±0.95	-	68.2±5.9	59.9±5.9						
E_{aJ}	$p = 0.43$	$p = 0.56$		$p = 0.12$	$p = 0.022$ (Temperature); $p = 0.045$ (Temperature^2)	$p = 0.99$		0.65	-	0.65
	-2.1±2.5	0.17±0.28	37.9±4.5	50.8±7.7	1±0.4 Temperature^2-11.9±3.8 Temperature					
T_{optA}	$p = 0.0058$			$p = 0.68$	$p = 0.97$	$p = 0.062$		0.37	-	0.37
	0.35±0.11	-	23.2±0.72	23.5±0.7						
b	$p = 0.86$			$p = 0.88$	$p = 0.046$	$p = 0.99$		0.53	-	0.53
	7.406e-05±4.380e-04	-	0.017±0.002	0.016±0.004	0.0013±0.0006					
A_{opt}	$p = 0.54$	$p = 0.029$		$p = 0.96$	$p = 0.029$	$p = 0.99$		0.63	-	0.63
	0.1±0.16	0.54±0.23	6.4±0.87	6.3±0.23	0.64±0.23					
A_g	$p = 0.76$	$p = 0.026$		$p = 0.99$	$p = 0.026$	$p = 0.99$		0.56	-	0.56
	0.047±0.15	0.53±0.22	6.4±0.82	6.4±0.21	0.57±0.21					
g_{s25}	$p = 0.00076$	-		$p = 0.72$	$p = 0.3$	$p = 0.99$		0.3	-	0.3
	0.003±0.001		0.049±0.007	0.046±0.008						
C_{i25}	$p = 0.76$			$p < 0.0001$	$p = 0.75$	$p = 0.035$		0.95	-	0.95
	0.77±2.57		205.3±16.1	502.2±16.1						

Thermal optimum of the maximum rate of Rubisco carboxylation - V_{cmax} (T_{optV}, °C); thermal optimum of the maximum rate of electron transport - J_{max} (T_{optJ}, °C); activation energy of V_{cmax} (E_{aV}; kJ mol^{-1}); activation energy of J_{max} (E_{aJ}; kJ mol^{-1}); thermal optimum of net photosynthesis (T_{optAn}, °C); and the *b* parameter (unitless); net photosynthesis rate at thermal optimum – A_{opt} (µmol m^{-2} s^{-1}); net photosynthesis rate estimated at monthly mean growth temperature (9 am – 3 pm) – A_g (µmol m^{-2} s^{-1}); stomatal conductance at a leaf temperature of 25 °C – g_{s25} (mol m^{-2} s^{-1}); intercellular CO_2 concentration at a leaf temperature of 25 °C - C_i (µmol mol^{-1}). A mixed-effects regression model was used to analyze the data where warming and elevated CO_2 treatment were the fixed effects, and the month in which the campaign was done was the random effect. Coefficient estimates ± standard error are given for each fixed effect (i.e., temperature and CO_2 treatments). The month intercept is given whenever the month included as a random intercept improved the model fit, however, for all statistical analyses the month had no significant effects on the model performance. Total (conditional) R^2 represents the total variation explained by the model and is portioned into variation explained by the fixed effects (marginal R^2) and random effect R^2. Significance threshold: $p < 0.05$. Mean±standard error.

Supplementary Table 2 | Parameter estimates for the adequate models explaining the effect of CO_2 treatment on the relationships between thermal optimum of net photosynthesis and its underlying biochemical temperature sensitivity parameters in tamarack and black spruce.

Tamarack									
Trait	x-variable	Intercept (aCO_2)	CO_2 treatment	x-variable*CO_2	Month intercept		Fixed effect (marginal R^2)	Random effect R^2	Total (Conditional) R^2
					Variance for random intercept (Month)	Residual variance			
T_{optA}	Slope (T_{optV})								
	$p = 0.0011$		$p < 0.0001$	$p = 0.057$	$p = 0.99$		0.72	-	0.72
	0.57±0.14	4.4±5.1	7.1±0.5						
T_{optA}	Slope (T_{optJ})								
	$p < 0.0001$		$p < 0.0001$	$p = 0.0569$	$p = 0.99$		0.79	-	0.79
	0.75±0.14	-0.62±4.8	2.4±0.5						
T_{optA}	Slope (E_{aV})								
	$p = 0.44$		$p = 0.0015$	$p = 0.29$	$p = 0.99$		0.48	-	0.48

	0.036±0.04	22.1±2.9	24.9±0.7						
T_{optA}	Slope (E_{aJ})								
	p = 0.67		p = 0.0015	p = 0.51	p = 0.73		0.46	-	0.46
	-0.025±0.06	25.4±2.4	28.1±0.7						

Black spruce									
Trait	x-variable	Intercept (aCO_2)	CO_2 treatment	x-variable*CO_2	Month intercept		Fixed effect (marginal R^2)	Random effect R^2	Total (Conditional) R^2
					Variance for random intercept (Month)	Residual variance			
	Slope (T_{optV})								
T_{optA}	p = 0.0108		p = 0.84	p = 0.49	p = 0.529		0.31	-	0.31
	0.52±0.2	7.4±6.1	7.5±0.7						
T_{optA}	Slope (T_{optJ})								
	p = 0.0026		p = 0.1695	p = 0.18	p = 0.15		0.42	-	0.42
	0.56±0.2	6.8±5.1	7.8±0.7						
T_{optA}	Slope (E_{aV})								
	p = 0.091		p = 0.36	p = 0.10	p = 0.61		0.16	-	0.16
	0.06±0.03	20.7±2.3	21.4±0.8						
T_{optA}	Slope (E_{aJ})								
	p = 0.55		p = 0.74	p = 0.65	p = 0.65		0.04	-	0.04
	-0.03±0.05	25.7±1.6	26±0.9						

Thermal optimum of net photosynthesis (T_{optA}, °C); thermal optimum of V_{cmax} (T_{optV}, °C); thermal optimum of J_{max} (T_{optJ}, °C); activation energy of V_{cmax} (E_{aV}; kJ mol^{-1}); activation energy of J_{max} (E_{aJ}; kJ mol^{-1}). A mixed-effects regression model was used to analyze the data where warming and elevated CO_2 treatment were the fixed effects, and the month in which the campaign was done was the random effect. Coefficient estimates ± standard error are given for each CO_2 treatment. The month intercept is given whenever the month included as a random intercept improved the model fit, however, for all statistical analyses the month had no significant effects on the model performance. Total (conditional) R^2 represents the total variation explained by the model and is portioned into variation explained by the fixed effects (marginal R^2) and random effect R^2. Significance threshold: $p < 0.05$. Mean±standard error. Mean±standard error.

Supplementary Table 3a | Statistical output of a t-Test (Two-sample assuming unequal variances) between the thermal optimum of net photosynthesis at measured intercellular CO_2 concentration (C_i) (T_{optA}) and the thermal optimum of net photosynthesis at a fixed C_i of 70 % (T_{opt70}) in tamarack and black spruce. The data were pooled across the two field campaigns (June and August). The statistical test was two-sided.

Tamarack

	T_{optA}	T_{opt70}
Mean	25.8	26.59
Variance	5.94	2.69
Observations	43	43
Hypothesized Mean Difference	0	
df	74	
t Stat	-1.75	
P(T<=t) one-tail	0.042	
t Critical one-tail	1.66	
P(T<=t) two-tail	0.083	
t Critical two-tail	1.99	

Black spruce

	T_{optA}	T_{opt70}
Mean	24.85	25.2
Variance	4.41	4.31
Observations	49	49
Hypothesized Mean Difference	0	
df	96	
t Stat	-0.83	
P(T<=t) one-tail	0.20	
t Critical one-tail	1.66	
P(T<=t) two-tail	0.41	
t Critical two-tail	1.98	

Supplementary Table 3b | Statistical output of a t-Test (Two-sample assuming unequal variances) between the thermal optimum of net photosynthesis (T_{optA}) and the thermal optimum of gross photosynthesis ($T_{optgross}$) both at measured intercellular CO_2 concentration (C_i) in tamarack and black spruce. The data were pooled across the two field campaigns (June and August). The statistical test was two-sided.

Tamarack

	T_{optA}	$T_{optgross}$
Mean	25.8	26.36
Variance	5.94	5.56
Observations	43	43
Hypothesized Mean Difference	0	
df	84	
t Stat	-1.07	
P(T<=t) one-tail	0.14	
t Critical one-tail	1.66	
P(T<=t) two-tail	0.29	
t Critical two-tail	1.99	

Black spruce

	T_{optA}	$T_{optgross}$
Mean	24.85	24.17
Variance	4.41	9.33
Observations	49	49
Hypothesized Mean Difference	0	
df	85	
t Stat	1.29	
P(T<=t) one-tail	0.1	
t Critical one-tail	1.66	
P(T<=t) two-tail	0.19	
t Critical two-tail	1.99	

Supplementary Table 4 | Summary report of a two-way ANOVA testing the effects of temperature and CO_2 treatments on the difference between mean daytime (9 am – 3 pm) growth temperature for the 10 days preceding each tree measurement and its respective T_{optA} ($\Delta MeanT_g$) for tamarack and black spruce. The analyses were done on pooled data across June and August campaigns. The statistical test was two-sided.

Tamarack

	DF	F-value	*p*-value
Temperature	4	6.9	0.0019
CO_2	1	9.9	0.0063
Temperature x CO_2	4	0.9	0.45
Residuals	16		

Black spruce

	DF	F-value	*p*-value
Temperature	4	19.7	<0.0001
CO_2	1	1.9	0.17
Temperature x CO_2	4	2.7	0.061
Residuals	18		

Supplementary Table 5 | Summary of an analysis of covariance (ANCOVA), where temperature is a covariate and CO_2 treatment a fixed factor, testing the relationship between net photosynthetic rates and monthly mean growth temperature during the growing season (June - September) of 2016 and 2017 in tamarack and black spruce. Mean±standard error.

Tamarack					
$A_{gTmean2016}$	Slope (Temperature and AC)	Intercept (AC)	CO_2 treatment	Temperature* CO_2	R^2
June	*p* = 0.23		*p* = 0.21	*p* = 0.36	0.33
	0.36±0.27	7.6±1.7	9.89±1.69		
July	*p* = 0.51		*p* = 0.13	*p* = 0.49	0.32
	0.19±0.27	7.9±1.67	10.7±1.65		
August	*p* = 0.44		*p* = 0.13	*p* = 0.47	0.24
	0.21±0.26	7.9±1.64	10.6±1.63		
September	*p* = 0.13		*p* = 0.35	*p* = 0.29	0.21
	0.47±0.28	7.15±1.69	8.8±1.68		
$A_{gTmean2017}$					
June	*p* = 0.41		*p* = 0.2	*p* = 0.34	0.32
	0.21±0.26	8.1±1.74	10.58±1.76		
July	*p* = 0.54		*p* = 0.12	*p* = 0.52	0.25
	0.17±0.27	7.9±1.66	10.74±1.64		
August	*p* = 0.3		*p* = 0.2	*p* = 0.41	0.27
	0.3±0.27	7.8±1.72	10.22±1.7		
September	*p* = 0.16		*p* = 0.33	*p* = 0.29	0.33
	0.42±0.27	7.29±1.73	9.06±1.71		

Black spruce					
$A_{gTmean2016}$	Slope (Temperature and AC)	Intercept (AC)	CO_2 treatment	Temperature* CO_2	R^2
June	*p* = 0.56		*p* = 0.86	*p* = 0.04	0.85
	0.087±0.14	6.4±0.76	6.92±1.06	0.6±0.19	
July	*p* = 0.96		*p* = 0.81	p = 0.053	0.81
	-0.0056±0.14	6.5±0.75	6.98±0.2	0.47±0.2	
August	*p* = 0.96		*p* = 0.78	*p* = 0.045	0.83

	0.0054±0.13	6.5±0.71	6.78±0.99	0.46±0.18	
September	p = 0.33		p = 0.94	p = 0.037	0.87
	0.15±0.15	6.09±0.76	6.17±1.06	0.69±0.2	
$A_{gTmean2017}$					
June	p = 0.85		p = 0.67	p = 0.076	0.78
	0.028±0.14	6.5±0.84	7.08±1.17	0.46±0.2	
July	p = 0.95		p = 0.79	p = 0.059	0.80
	-0.0084±0.14	6.47±0.76	6.75±1.06	0.45±0.19	
August	p = 0.65		p = 0.87	p = 0.048	0.83
	0.06±0.14	6.46±0.78	6.64±1.1	0.56±0.2	
September	p = 0.36		p = 0.99	p = 0.034	0.86
	0.13±0.14	6.16±0.76	6.16±1.07	0.66±0.19	

Supplementary Table 6 | Summary of comparison between models with interaction of the main treatments effects and models with only main treatments effects using Akaike Information Criteria (AICc) on leaf traits for tamarack and black spruce.

Tamarack												
Model	T_{optAn}		T_{optV}		T_{optJ}		E_{aV}		E_{aJ}		b	
	AICc	ΔAICc	AICc	ΔAICc	AICc	ΔAICc	AICc	ΔAICc	AICc	ΔAICc	AICc	ΔAICc
Temperature*CO_2	80.9	2.4	88.2	3.4	84.1	3.3	153.8	2.6	145.6	3.7	-122.8	3.1
Temperature+CO_2	78.5	**0**	84.8	**0.0**	80.8	**0.0**	151.2	**0.0**	141.9	**0.0**	-125.9	**0**
Black spruce												
Temperature*CO_2	85.2	3.6	83.3	9	69.0	1	169.9	2.4	145.3	0.0	-152.3	1.5
Temperature+CO_2	81.6	**0.0**	74.3	**0.0**	70.0	**0.0**	167.5	**0.0**	146.4	**1.1**	-150.8	**0**

Tamarack				
Model	A_{opt}		A_g	
	AICc	ΔAICc	AICc	ΔAICc
Temperature*CO_2	112.2	2.9	110.4	**0**
Temperature+CO_2	109.3	**0**	107.7	2.7
Black spruce				
Temperature*CO_2	84.6	**0**	82.5	**0**
Temperature+CO_2	87.1	2.5	85.2	2.7

For each trait the optimal model is bolded, and the summary output of the model is presented in the Supplementary Table 1. Thermal optimum of V_{cmax} (T_{optV}, °C); thermal optimum of J_{max} (T_{optJ}, °C); activation energy of V_{cmax} (E_{aV}; kJ mol^{-1}); activation energy of J_{max}

(E_{aJ}; kJ mol^{-1}); thermal optimum of net photosynthesis (T_{optAn}, °C); and the b parameter (b); net photosynthesis rate at thermal optimum – A_{opt} (μmol m^{-2} s^{-1}); net photosynthesis rate modeled at monthly mean growth temperature (9 am – 3 pm) – A_g (μmol m^{-2} s^{-1}).

Supplementary Table 7 | Summary of model comparisons of the relationships between the thermal optimum of net photosynthesis at measured intercellular CO_2 concentration (C_i) and other photosynthetic temperature sensitivity parameters, with CO_2 treatment as a covariate in tamarack and black spruce. Akaike Information Criteria (AICc) are presented for models with interaction and with only main effects.

Tamarack		
Model	T_{optAn}	
	AICc	ΔAICc
T_{optV}*CO_2	86.5	3
T_{optV}+CO_2	83.5	**0.0**
T_{optJ}*CO_2	65.1	**0**
T_{optJ}+CO_2	66.2	1.1
E_{aV}*CO_2	86.5	2.3
E_{aV}+CO_2	84.2	**0.0**
E_{aJ}*CO_2	87.8	3
E_{aJ}+CO_2	84.8	**0.0**

Black spruce		
Model	T_{optAn}	
	AICc	ΔAICc
T_{optV}*CO_2	86.5	3.3
T_{optV}+CO_2	83.5	**0.0**
T_{optJ}*CO_2	81.2	1.3
T_{optJ}+CO_2	79.9	**0.0**
E_{aV}*CO_2	87.6	0.0
E_{aV}+CO_2	87.4	**0.2**
E_{aJ}*CO_2	90.0	3.5
E_{aJ}+CO_2	86.5	**0.0**

For each trait the optimal model is bolded, and the summary output of the model is presented in the Supplementary Table 2. Thermal optimum of net photosynthesis (T_{optA}, °C); thermal optimum of V_{cmax} (T_{optV}, °C); thermal optimum of J_{max} (T_{optJ}, °C); activation energy of V_{cmax} (E_{aV}; kJ mol^{-1}); activation energy of J_{max} (E_{aJ}; kJ mol^{-1}).
