# Supplementary material for: Boreal conifers maintain carbon uptake with warming despite failure to track optimal temperatures
Source: Nat Commun. 2023 Aug 3;14:4667. doi: 10.1038/s41467-023-40248-3 (PMC10400668; doi:10.1038/s41467-023-40248-3)
Supplement: Supplementary file 3 — Reporting Summary [file 41467_2023_40248_MOESM3_ESM.pdf]

nature portfolio

Corresponding author(s): Mirindi Eric Dusenge
Danielle A Way

Last updated by author(s): July 2nd, 2023

Reporting Summary

Nature Portfolio wishes to improve the reproducibility of the work that we publish. This form provides structure for consistency and transparency in reporting. For further information on Nature Portfolio policies, see our Editorial Policies and the Editorial Policy Checklist.

Statistics

For all statistical analyses, confirm that the following items are present in the figure legend, table legend, main text, or Methods section.

n/a	Confirmed	
☐	☒	The exact sample size (*n*) for each experimental group/condition, given as a discrete number and unit of measurement
☐	☒	A statement on whether measurements were taken from distinct samples or whether the same sample was measured repeatedly
☐	☒	The statistical test(s) used AND whether they are one- or two-sided<br>*Only common tests should be described solely by name; describe more complex techniques in the Methods section.*
☐	☒	A description of all covariates tested
☐	☒	A description of any assumptions or corrections, such as tests of normality and adjustment for multiple comparisons
☐	☒	A full description of the statistical parameters including central tendency (e.g. means) or other basic estimates (e.g. regression coefficient) AND variation (e.g. standard deviation) or associated estimates of uncertainty (e.g. confidence intervals)
☐	☒	For null hypothesis testing, the test statistic (e.g. *F*, *t*, *r*) with confidence intervals, effect sizes, degrees of freedom and *P* value noted<br>*Give P values as exact values whenever suitable.*
☒	☐	For Bayesian analysis, information on the choice of priors and Markov chain Monte Carlo settings
☐	☒	For hierarchical and complex designs, identification of the appropriate level for tests and full reporting of outcomes
☒	☐	Estimates of effect sizes (e.g. Cohen's *d*, Pearson's *r*), indicating how they were calculated

Our web collection on statistics for biologists contains articles on many of the points above.

Software and code

Policy information about availability of computer code

Data collection	We used the Li-COR 6400 XT (LI-COR Biosciences, Lincoln, NE, USA) portable photosynthesis systems, version 6.3.4.
Data analysis	To analyze the data, we used R 3.6.1. software (R Core Team, 2019). We also used following open-source R packages for statistical analyses: plantecophys 1.4-6, nlme 3.1-162, and AICcmodavg 2.3.2. We also used following softwares: Excel 16.74 and ImageJ 1.51.

For manuscripts utilizing custom algorithms or software that are central to the research but not yet described in published literature, software must be made available to editors and reviewers. We strongly encourage code deposition in a community repository (e.g. GitHub). See the Nature Portfolio guidelines for submitting code & software for further information.

Data

Policy information about availability of data

All manuscripts must include a data availability statement. This statement should provide the following information, where applicable:

- Accession codes, unique identifiers, or web links for publicly available datasets
- A description of any restrictions on data availability
- For clinical datasets or third party data, please ensure that the statement adheres to our policy

The raw and processed (i.e., mean values used to generate each figure in the paper) photosynthesis data generated in this study have been deposited in the figshare database and can be accessed at https://doi.org/10.6084/m9.figshare.22645030. The complete leaf gas exchange data, including the data used in this paper, are also available through the SPRUCE project website at https://doi.org/10.25581/spruce.056/1455138.

Human research participants

Policy information about studies involving human research participants and Sex and Gender in Research.

Reporting on sex and gender	n/a
Population characteristics	n/a
Recruitment	n/a
Ethics oversight	n/a

Note that full information on the approval of the study protocol must also be provided in the manuscript.

Field-specific reporting

Please select the one below that is the best fit for your research. If you are not sure, read the appropriate sections before making your selection.

☐ Life sciences ☐ Behavioural & social sciences ☒ Ecological, evolutionary & environmental sciences

For a reference copy of the document with all sections, see nature.com/documents/nr-reporting-summary-flat.pdf

Ecological, evolutionary & environmental sciences study design

All studies must disclose on these points even when the disclosure is negative.

Study description	This study was conducted at the Oak Ridge National Laboratory's SPRUCE (Spruce and Peatland Responses Under Changing Environments) project site at the U.S. Forest Service's Marcell Experimental Forest, in Minnesota, USA (47°30.476′ N; 93°27.162′ W). The details of the study site and experimental design are provided in recent studies from this experiment 50,54–56. But briefly, this forest grows naturally in a bog located at the southern limit of the boreal peatland forests. The forest is approximately 50 years old as it regenerated following canopy tree removal in 1969 and 197452. The dominant canopy species is Picea mariana (Mill.) B.S.P. (black spruce) mixed with less abundant Larix laricina (Du Roi) K. Koch (tamarack). The understory vegetation is dominated by ericaceous shrubs Rhododendron groenlandicum (Oeder) Kron & Judd and Chamaedaphne calyculata (L.) Moench. The experiment comprises five temperature treatments (ambient or +0, which serves also as the control, +2.25, +4.5, +6.75, and +9 °C above the ambient) established in a regression-based design53. This experiment uses 10 large octagonal open-top enclosures with an interior surface area of 114.8 m2, and a sampling area of 66.4 m2. Five enclosures have an ambient-CO2 atmosphere, while the other five have an elevated CO2 atmosphere varying between + 430 and 500 ppm above the ambient. The heating treatments started August 15, 2015, and CO2 treatments were initiated a year later, on June 15, 2016.
Research sample	We studied the two mixed-age (up to ~45 years old) canopy tree species at SPRUCE, Picea mariana (Mill.) B.S.P. (black spruce) and Larix laricina (Du Roi) K. Koch (tamarack). For black spruce, one branchlet for each, randomly selected tree and in each plot was harvested and one-year needle cohorts (i.e., developed in growth season of 2016) from each branch was measured. For tamarack, fully expanded current year foliage was used. In the June field campaign, three trees in each plot were randomly sampled, while in the August campaign, only two trees were used. For tamarack, we used the same number of branchlets from different trees in each plot, except in one plot (in ambient CO2 and +0) where only one tamarack tree was available to be sampled. All measurements were made on sun-exposed branchlets cut using a pruning pole. After cutting, branchlets were put in water, and recut under water to avoid xylem transport disruption and stomatal closure. The branches were harvested between 4 – 5 am of the measurement day, placed in water bottles inside a plastic cooler, and transported from the field site in Marcell, Minnesota to the walk-in growth chambers at the University of Minnesota in St. Paul, where the measurements were conducted. The branchlets were re-cut again before starting the measurements. The effect of cutting and the time lag between cutting and gas exchange measurements had no effect on stomatal conductance in these conifers.
Sampling strategy	For the two studied species, three trees in each experimental plot were randomly sampled in the June campaign, while in the August campaign, only two trees were used. For one of the species (tamarack), there was only one tree in one of the plot (in ambient CO2 and +0) that could be sampled. The sample size is enough, since our study use a regression-based experimental design with five levels of temperature treatments.
Data collection	The data were collected using seven portable photosynthesis systems (Li-COR 6400 XT, 6400-18 RGB light source, and 6400-22 opaque conifer chamber; LI-COR Biosciences, Lincoln, NE, USA). These instruments, were operated by six skilled persons. The preparation checklist was conducted everyday and checked across all the instruments to minimize instrument-specific background noise.
Timing and spatial scale	We collected the data in two field campaigns, one done in June (18 to 30th) and another in August (13th to 23rd) 2017. We conducted these two campaigns to ensure that there might not be a seasonal effect in our results. Such effect was not found on the responses of the traits reported in this study. All these data were collected from the trees at the SPRUCE experiment.
Data exclusions	We conducted a quality check on all the raw data collected. The criteria used were to check for any negative value of the intercellular CO2 concentration (Ci) and stomatal conductance (gs) and exclude them. No tree measurement was found to violate this criteria. However, during fitting of the Farquhar et al. 1980 model, the Arrhenius function, and the second order polynomial function on

	temperature responses of net photosynthesis, some parameter values for few trees (six values in total) were excluded based on the visual inspection of whether the model fitting/outputs accurately represented the observations.
Reproducibility	We have provided access to the raw and processed data used in this study (https://doi.org/10.6084/m9.figshare.22645030), and we have provided in details all the parameterization used to derive parameters reported in this study.
Randomization	Experimental plots and infrastructure in support of the SPRUCE Whole Ecosystem Warming study were established on the S1-Bog of the Marcell Experimental Forest. Three ~ 100 adjacent and parallel transect boardwalks were built for accessing 17 octagonal permanent plots over the southern half of the 8.1 ha bog. In all, 10 of the permanent plots were randomly assigned to the following warming treatments: two fully constructed control plots with no energy added (henceforth simply control plots), and two plots each to be managed as + 2.25, + 4.5, + 6.75 and + 9 degree Celsius warming plots. In half of each warming level, elevated CO2 of +500 ppm treatment was randomly assigned. During the two measurements field campaigns, trees sampled for this study were randomly chosen, which resulted in measuring different trees in each plot between the June and the August campaign.
Blinding	The team that sampled the branches used in this experiment was not the same that measured them with gas exchange equipments. Furthermore, only the codes representing plot number was indicated on the branch, which did not directly indicate in which treatment the sample was taken from.

Did the study involve field work? ☒ Yes ☐ No

Field work, collection and transport

Field conditions	The information on the environmental field conditions at this experiment is freely available from the project climate data link: https://sprucedata.ornl.gov/vdv.php/historical/156
Location	The experiment is located at the U.S. Forest Service's Marcell Experimental Forest, in Minnesota, USA (47°30.476' N; 93°27.162' W).
Access & import/export	No sample material was exported out of the country (USA).
Disturbance	To minimize the destructive sampling at the experiment, we made sure to determined before-hand the adequate number of samples needed for a robust statistical analysis. This was important to consider since the experiment will run for 10 years (at least).

Reporting for specific materials, systems and methods

We require information from authors about some types of materials, experimental systems and methods used in many studies. Here, indicate whether each material, system or method listed is relevant to your study. If you are not sure if a list item applies to your research, read the appropriate section before selecting a response.

Materials & experimental systems

n/a	Involved in the study	
☒	☐	Antibodies
☒	☐	Eukaryotic cell lines
☒	☐	Palaeontology and archaeology
☒	☐	Animals and other organisms
☒	☐	Clinical data
☒	☐	Dual use research of concern

Methods

n/a	Involved in the study	
☒	☐	ChIP-seq
☒	☐	Flow cytometry
☒	☐	MRI-based neuroimaging
